# Diabetes and the brain: issues and unmet needs

**DOI:** 10.1007/s10072-014-1797-2

**Published:** 2014-04-29

**Authors:** Natan M. Bornstein, Michael Brainin, Alla Guekht, Ingmar Skoog, Amos D. Korczyn

**Affiliations:** 1 Department of Neurology, Tel Aviv Medical School, Tel Aviv University, Ramat Aviv, 69978 Tel Aviv, Israel; 2 Department of Clinical Neurosciences and Preventive Medicine, Danube University Krems, Krems, Austria; 3 Department of Neurology and Neurosurgery, Russian National Research Medical University, Moscow City Hospital No. 8 for Neuropsychiatry, Moscow, Russia; 4 Sahlgrenska Academy, University of Gothenburg, Gothenburg, Sweden

**Keywords:** Diabetes, Cognitive impairment, Vascular dementia, Stroke

## Abstract

Diabetes mellitus (DM) is associated with an increased risk of mild cognitive impairment, dementia and stroke. The association between DM and dementia appears to be stronger for vascular cognitive impairment than for Alzheimer’s disease, suggesting cerebrovascular disease may be an important factor in cognitive impairment in DM. Although the exact mechanisms by which DM affects the brain remain unclear, changes to brain vasculature, disturbances of cerebral insulin signaling, insulin resistance, glucose toxicity, oxidative stress, accumulation of advanced glycation end products, hypoglycemic episodes, and alterations in amyloid metabolism may all be involved. Cognitive impairment and dementia associated with DM may also be mediated via vascular risk factors, in particular brain ischemia, the occurrence of which can have an additive or synergistic effect with concomitant neurodegenerative processes. To date, no drug has been approved for the treatment of vascular dementia and there are no specific pharmacological treatments for preventing or reducing cognitive decline in patients with DM. Most focus has been on tighter management of vascular risk factors, although evidence of reduced cognitive decline through reducing blood pressure, lipid-lowering or tighter glycemic control is inconclusive. Tailored, multimodal therapies may be required to reduce the risk of cognitive dysfunction and decline in patients with DM. The use of pleiotropic drugs with multimodal mechanisms of action (e.g., cerebrolysin, Actovegin) may have a role in the treatment of cognitive dysfunction and their use may warrant further investigation in diabetic populations.

## Introduction

Diabetes mellitus (DM) is associated with an increased risk of mild cognitive impairment, dementia, and stroke [1]. DM is likely to become an increasingly important contributory factor in dementia, especially given an estimated global population of 552 million affected individuals by 2030 [2].

Vascular brain pathology underlying cognitive decline is heterogeneous and can involve a variety of processes leading to acute or chronic ischemia or a combination of both. Although Alzheimer’s disease (AD) is generally considered the most frequent dementia diagnosis, vascular cognitive decline may be more common than previously believed. However, it can be difficult to distinguish between the two and most patients, particularly in old age, will have mixed dementia [3]. Several cohort studies have shown mixed pathology on autopsy in the majority of dementia patients, including AD changes (e.g., amyloid-beta plaques) and cerebrovascular lesions (infarcts, lacunas, microbleeds and white matter lesions) [4].

## Diabetes and cognitive function

Several studies have shown that DM is a risk factor for cognitive impairment and dementia [5, 6]. These mostly focus on type 2 rather than type 1 DM, which accounts for the majority of DM patients (at least 90 %). Cross-sectional studies have generally shown worse cognitive performance in patients with DM compared with matched controls [7]. Longitudinal studies have also reported accelerated cognitive decline in patients with DM [8, 9]. Two studies have recently reported that higher glucose levels may be a risk factor for cognitive impairment or dementia even among persons without DM [10, 11]. Modest cognitive decrements are already present in patients with early-stage type 2 DM [12] and metabolic syndrome has been reported to affect cognition and raise the risk of dementia [13]. However, evidence supporting a causal association between DM and cognitive impairment is mixed.

The association between DM and dementia appears to be stronger for vascular cognitive impairment than for AD. A recent meta-analysis reported that DM was associated with an increased relative risk of 1.2 for mild cognitive impairment, 1.5 for AD and 2.5 for vascular dementia [14]. Elderly patients with DM have also been reported to have a reduced amyloid-beta load and more cerebral infarcts versus non-diabetics [15].

DM may be associated with modest cognitive decrements in non-demented individuals that progress only slowly over time, causing subtle changes to self-esteem, mood, and wellbeing. DM may also be associated with an increased risk of more severe cognitive deficits and dementia in certain patients. These two processes may reflect a continuum with modest impairment at an earlier stage; however, a distinction between these two types has been noted with regard to age groups and trajectories of development, and it has been suggested that these may reflect separate processes [16]. If so, these two processes may be associated with different risk factors and potentially require different treatments.

**Fig. 1:**
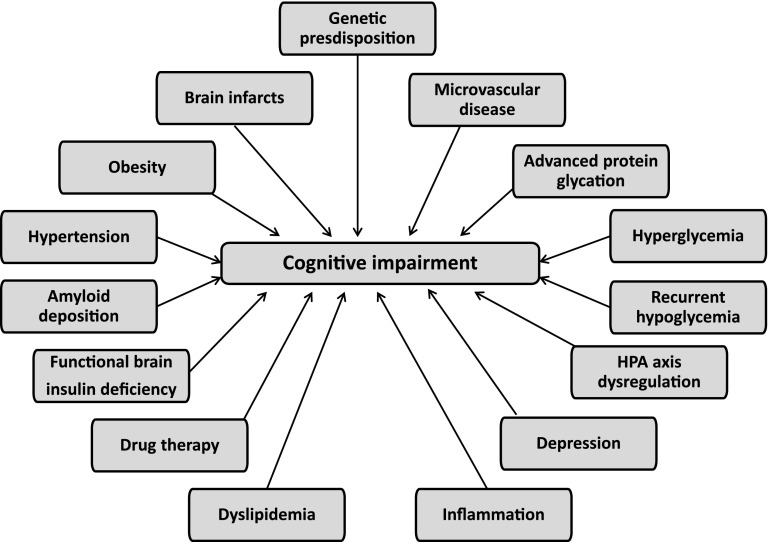
Potential causes of cognitive impairment in type 2 diabetes. Adapted from [17]

A wide range of metabolic and vascular disturbances have been implicated in the pathophysiology of cognitive impairment (Fig. 1) [17]. The exact mechanisms by which DM affects the brain remain unclear but probably involve both cerebrovascular and neurodegenerative changes. Changes to brain vasculature, disturbances of cerebral insulin signaling, insulin resistance, glucose toxicity, oxidative stress, accumulation of advanced glycation end products, hypoglycemic episodes, and alterations in amyloid metabolism may all be involved. Neuroimaging studies have shown structural changes to the brain in patients with DM, with magnetic resonance imaging (MRI) studies showing an association between DM and silent and asymptomatic brain infarcts [18]. DM has also been reported to be a risk factor for white matter lesion (WML) progression [19], although this relationship is less consistent.

## Diabetes and acute ischemic stroke

Cognitive impairment and dementia associated with DM may be mediated via ischemic stroke, which can have an additive or synergistic effect with concomitant neurodegenerative lesions. Patients with DM are well recognized as being at increased risk of stroke. In a meta-analysis involving almost 700,000 patients, DM is more than doubled the risk of ischemic stroke after adjusting for body mass index, blood pressure, lipids, and other risk factors (hazard ratio 2.27; 95 % CI 1.95–2.65) [20]. DM is also associated with worse outcomes in stroke patients, in particular increased mortality [21].

There is a high rate of previously undiagnosed DM in acute stroke patients. In one cohort of 238 acute stroke patients, 36 % had DM, including 16 % with newly diagnosed DM. An additional 24 % had impaired glucose tolerance or abnormally high fasting glucose [22]. The proportion of acute stroke patients with previously unknown DM rather than transient stress hyperglycemia may be higher than is often thought, especially since criteria for defining DM in stroke trials are typically a history of DM or intake of anti-diabetes medications [23].

Hyperglycemia during the acute phase of stroke is associated with worse short-term outcomes. In a systematic review, relative risk of in-hospital or 30-day mortality after an ischemic stroke was 3.3 in hyperglycemic patients without known DM and 2.0 in those with known DM when compared to patients with normoglycemia [24]. In an analysis of 268 patients with a non-lacunar stroke, admission hyperglycemia was negatively correlated with the degree of neurological improvement at 24 h in reperfused but not non-reperfused recombinant tissue plasminogen activator (rt-PA)-treated patients [25], suggesting the deleterious effect of hyperglycemia on infarct growth may be related to whether or not reperfusion occurs. However, in another trial, higher admission glucose levels were associated with decreased likelihood of neurological improvements and increased risk of symptomatic intracerebral hemorrhage, regardless of rt-PA treatment [26].

**Fig. 2:**
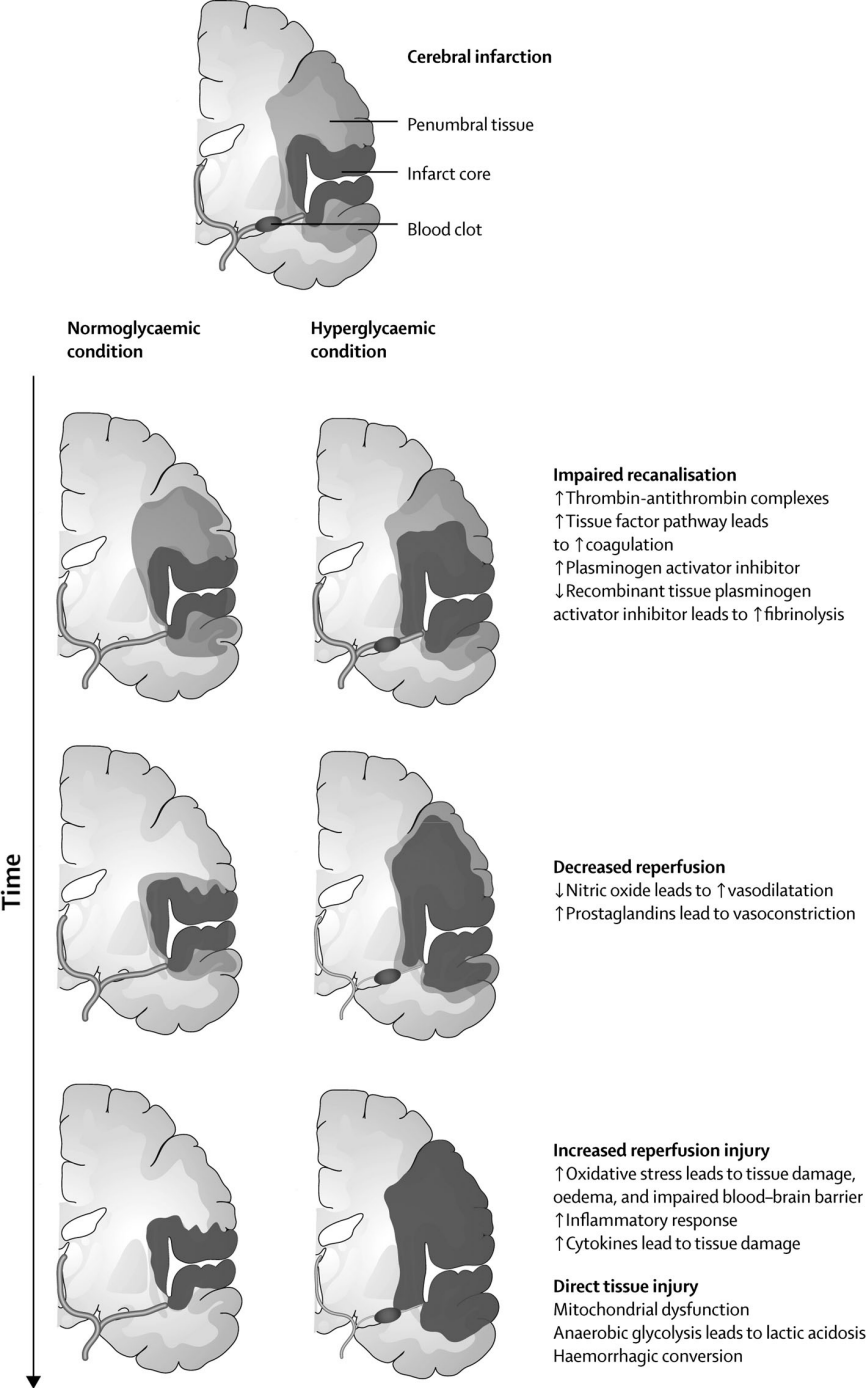
Potential effects of hyperglycaemia over time on pathological development of cerebral infarction (reproduced with permission from [27])

Several mechanisms have been suggested by which hyperglycemia may have a deleterious effect in ischemic stroke, including impaired recanalization and increased reperfusion injury (Fig. 2) [27]. The association between hyperglycemia and poor outcome after stroke is mainly related to large-vessel infarction and is less evident in lacunar stroke [28]. This may be because hyperglycemia reduces salvage of the penumbra, the ischemic area that can potentially recover if adequate reperfusion is administered within hours of stroke onset. A penumbra is not usually present in lacunar stroke.

The deleterious effect of hyperglycemia on stroke outcomes raises the question of whether glucose-lowering treatment during the acute phase of stroke may be beneficial. In other critically ill (non-stroke) patients with hyperglycemia, initial studies suggested that intensive insulin therapy could be beneficial [29]. However, later studies failed to confirm these findings. In the NICE-SUGAR study, intensive glucose control increased mortality among adults in intensive care, with a blood glucose target of 180 mg/dL or less resulting in lower mortality than a target of 81–108 mg/dL [30]. Moreover, more intensive glucose-lowering treatment may be associated with an increased risk of severe hypoglycemia [31].

There is no evidence to date that glucose-lowering improves clinical outcomes in acute stroke. In the GIST-UK study, patients presenting within 24 h of stroke onset randomized to variable-dose glucose–potassium–insulin had significantly reduced plasma glucose concentration and systolic blood pressure but no significant reduction in mortality at 90 days compared with the control group [32]. However, it should be noted that the trial was underpowered, over 20 % of patients had lacunar stroke and glucose levels during the 24-h treatment period were only 0.57 mmol/L lower in the intensive treatment group. In a pilot study in which patients were randomized to intensive insulin (target glucose <7.2 mmol/L) or standard insulin treatment (target glucose <11.1 mmol/L), clinical outcomes were considered somewhat better with intensive therapy, although differences between groups were not significant [33]. A definitive phase III trial (SHINE) is currently underway to compare standard of care glucose control to an intensive level of control in hyperglycemic acute ischemic stroke patients [34].

## Treatment options for preventing or reducing cognitive decline

To date, there are no pharmacological treatments for preventing or reducing cognitive decline in patients with DM. Most focus has been on tighter management of vascular risk factors to help ameliorate cognitive decline. Anti-hypertensive therapy has been reported to reduce dementia risk in the general population, although results from randomized, controlled studies are inconclusive [35]. In the PROGRESS trial of 6,105 individuals with prior stroke or transient ischemic attack, cognitive decline was significantly less with perindopril (with or without indapamide) compared with placebo, and there was a non-significant decrease in incident dementia [36]. Similarly, the HYVET-Cog study on indapamide with or without perindopril showed a non-significant effect on incident dementia of 0.86 (95 % CI 0.67–1.09) [37]. Other studies reported no benefit of anti-hypertensive treatment on cognitive performance or dementia incidence [38, 39]. The efficacy of lipid-lowering therapy in reducing cognitive decline is also not proven, with no effect seen with simvastatin [40] or pravastatin [41]. Several methodological issues may hinder the ability to demonstrate reduced cognitive decline through vascular risk factor reduction, including patients being relatively young with low incidence of cognitive dysfunction, insufficient follow-up, high dropout rates due to cognitive impairment, and additional risk factor intervention in the placebo/control group [35].

**Fig. 3:**
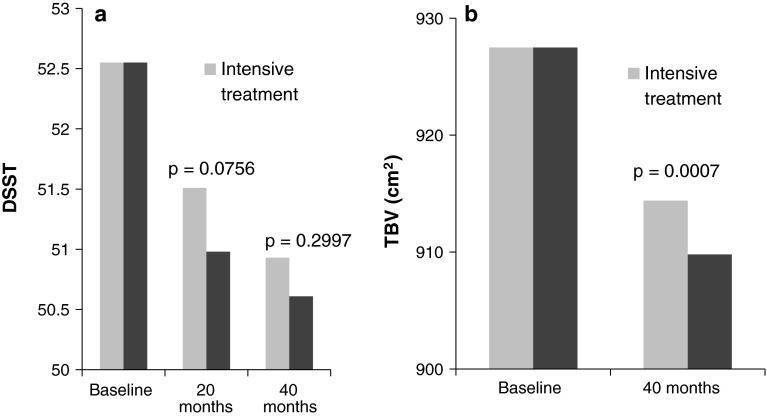
Outcomes in the ACCORD-MIND study [46]. **a** Digit symbol substitution test (DSST). **b** Total brain volume

In patients with DM, the effect of tighter glycemic control on cognitive function is inconsistent. Some studies have reported a benefit of improved control on cognitive decline [42, 43] while others have shown no difference [44]. In the ADDITION study, cognitive decline in patients with screening-detected type 2 DM did not differ between intensive multifactorial treatment and routine care after 6 years [45]. Moreover, cognitive decline in both groups was within the range observed in a control group of non-diabetic study participants. In the largest trial reported to date, the ACCORD-MIND study, cognitive function at 40 months was not improved with intensive (HbA1c <6.0 %) compared with standard glycemic control (HbA1c 7.0–7.9 %) [46]. However, decline in total brain volume was significantly reduced in the intensive therapy group (Fig. 3). Structural changes in the brain may occur before cognitive differences between groups are apparent and longer-term follow-up may be needed to detect a benefit of more intensive control. It has also been suggested that mean cognitive performance was relatively stable over time in both groups, leaving little room for a treatment effect [6]. However, given the increased mortality observed in patients with intensive treatment, tighter glycemic control is not recommended to reduce the adverse effects of DM on the brain. Moreover, a history of severe hypoglycemic episodes has also been associated with increased dementia, suggesting any benefits of tighter control may need to be balanced against a higher hypoglycemia risk [47].

The effects of oral anti-diabetic drugs in the prevention and treatment of vascular cognitive impairment are still to be clarified. Some studies initially suggested that thiazolidinediones may have a beneficial impact on cognitive function in patients with AD [48]. However, larger, more robust trials of rosiglitazone failed to show any benefit [49]. Data from the ACCORD-MIND study suggested that treatment with rosiglitazone was associated with increased cognitive decline after 40 months [50], although these results may have been confounded by unexplained differences between patients. No relationship between insulin use and cognitive function was observed. Metformin has been associated with an increase in amyloid peptides in neuronal cultures, which raises the possibility that metformin may accelerate clinical manifestations of AD in patients with type 2 DM [51]. In a recent retrospective study, metformin use was associated with worse cognitive performance among patients with DM [52]. However, animal studies have suggested metformin may ameliorate AD-like biochemical changes [53].

No drug has yet been approved for the treatment of vascular dementia. AD treatments, such as the cholinesterase inhibitors donepezil and galantamine, have shown some cognitive benefits in clinical trials, but effects on global and functional efficacy were less consistent [54, 55]. No clear benefit with NMDA receptor antagonists (e.g., memantine) has been shown in vascular dementia. The small cognitive improvements observed in some patients with these treatments may actually result from an effect on co-existing AD [56].

Pleiotropic drugs with multimodal mechanisms of action (e.g., cerebrolysin) have shown some potential, although these findings need to be confirmed [57]. Another agent with pleiotropic neuroprotective and metabolic effects is Actovegin. The effects of Actovegin include increased oxygen utilisation and uptake, improved glucose metabolism, increased neuron survival, inhibition of poly(ADP-ribose) polymerase activity, reduced oxidative stress, activation of NF-κB, and reduced apoptosis [58]. In a randomized, double-blind, placebo-controlled trial of 567 patients, Actovegin was associated with improvements in symptoms of diabetic polyneuropathy [59]. Other studies have suggested a beneficial effect of Actovegin on cognitive function, currently being further investigated in a randomized, controlled trial [60]. Given its pleiotropic neuroprotective and metabolic actions, the effect of Actovegin on cognitive function in patients with DM may be worthy of further investigation.

## Conclusions

Cognitive impairment and dementia in patients with DM is likely to become an increasing problem. However, to date, there are no specific treatments for cognitive impairment or the prevention of further cognitive decline in the general population or specifically patients with DM. Tailored, multimodal therapies may be required to reduce the risk of cognitive dysfunction and decline in patients with DM. The use of pleiotropic drugs may have a role in the treatment of cognitive dysfunction and their use may warrant further investigation in diabetic populations.
